# A deep look into the storm: Israeli multi-center experience of coronavirus disease 2019 (COVID-19) in patients with autoimmune inflammatory rheumatic diseases before and after vaccinations

**DOI:** 10.3389/fimmu.2023.1064839

**Published:** 2023-03-13

**Authors:** Fadi Kharouf, Tali Eviatar, Maya Braun, Elisheva Pokroy-Shapira, Michal Brodavka, Yair Zloof, Nancy Agmon-Levin, Kochava Toledano, Shirly Oren, Merav Lidar, Devy Zisman, Yonit Tavor, Mirit Amit-Vazina, Firas Sabbah, Gabriel S. Breuer, Amir Dagan, Rima Beshara-Garzuzi, Doron Markovits, Muna Elias, Joy Feld, Oshrat Tayer-Shifman, Tal Gazitt, Tatiana Reitblatt, Limor Rubin, Amir Haddad, Sami Giryes, Daphna Paran, Hagit Peleg, Yair Molad, Ori Elkayam, Dror Mevorach, Alexandra Balbir-Gurman, Yolanda Braun-Moscovici

**Affiliations:** ^1^ The Department of Medicine, Rheumatology Unit and Rare Disease Research Center, Hadassah Medical Center, Jerusalem, Israel; ^2^ The Faculty of Medicine, Hebrew University of Jerusalem, Jerusalem, Israel; ^3^ Rheumatology Department, Sourasky Medical Center, Tel Aviv, Israel; ^4^ Sackler Faculty of Medicine, Tel Aviv University, Tel Aviv, Israel; ^5^ Institute of Rheumatology, Rabin Medical Center, Beilinson Hospital, Petach Tikva, Israel; ^6^ Rheumatology Unit, Sheba Medical Center, Ramat Gan, Israel; ^7^ Clinical Immunology, Angioedema and Allergy Unit, Sheba Medical Center, Ramat Gan, Israel; ^8^ Rheumatology Institute, Rambam Health Care Campus, Haifa, Israel; ^9^ Rappaport Faculty of Medicine, Israeli Institute of Technology-Technion, Haifa, Israel; ^10^ Rheumatology Unit, Carmel Medical Center, Haifa, Israel; ^11^ Rheumatology Service, Shamir Medical Center, Be’er Ya’akov, Israel; ^12^ Rheumatology Service, Baruch Padeh Medical Center, Tiberias, Israel; ^13^ Rheumatology Unit, Shaare Tzedek Medical Center, Jerusalem, Israel; ^14^ Rheumatology Clinic, Assuta Ashdod Hospital, Ashdod, Israel; ^15^ Rheumatology Unit, Meir Medical Center, Kfar Saba, Israel; ^16^ Barzilai Medical Center, Ben Gurion University, Ashkelon, Israel; ^17^ Allergy and Clinical Immunology Unit, Department of medicine, Hadassah Medical Center, Jerusalem, Israel

**Keywords:** AIIRD, autoimmune inflammatory rheumatic disease, COVID-19, coronavirus disease 2019, outbreaks, SARS-CoV-2, severe acute respiratory syndrome coronavirus-2, vaccination

## Abstract

**Objective:**

We aimed to characterize the course of COVID-19 in autoimmune inflammatory rheumatic disease (AIIRD) patients in Israel, taking into consideration several remarkable aspects, including the outcomes of the different outbreaks, the effect of vaccination campaigns, and AIIRD activity post-recovery.

**Methods:**

We established a national registry of AIIRD patients diagnosed with COVID-19, including demographic data, AIIRD diagnosis, duration and systemic involvement, comorbidities, date of COVID-19 diagnosis, clinical course, and dates of vaccinations. COVID-19 was diagnosed by a positive SARS-CoV-2 polymerase chain reaction.

**Results:**

Israel experienced 4 outbreaks of COVID-19 until 30.11.2021. The first three outbreaks (1.3.2020 – 30.4.2021) comprised 298 AIIRD patients. 64.9% had a mild disease and 24.2% had a severe course; 161 (53.3%) patients were hospitalized, 27 (8.9%) died. The 4^th^ outbreak (delta variant), starting 6 months after the beginning of the vaccination campaign comprised 110 patients. Despite similar demographic and clinical characteristics, a smaller proportion of AIIRD patients had negative outcomes as compared to the first 3 outbreaks, with regards to severity (16 patients,14.5%), hospitalization (29 patients, 26.4%) and death (7 patients, 6.4%). COVID-19 did not seem to influence the AIIRD activity 1-3 months post-recovery.

**Conclusions:**

COVID-19 is more severe and has an increased mortality in active AIIRD patients with systemic involvement, older age and comorbidities. Vaccination with 3 doses of the mRNA vaccine against SARS-CoV-2 protected from severe COVID-19, hospitalization and death during the 4^th^ outbreak. The pattern of spread of COVID-19 in AIIRD patients was similar to the general population.

## Introduction

Coronavirus disease 2019 (COVID-19) has impacted every aspect in medicine, and rheumatology was not spared. Concerns were raised whether patients with autoimmune inflammatory rheumatic diseases (AIIRDs) are more vulnerable to COVID-19, and the role of immunosuppressive therapies was not clearly defined. Over the past 2 years, numerous studies regarding COVID-19 among AIIRD patients, including data from national and multinational registries, were published (1–13).

We learned from the studies performed that “classic” risk factors such as age older than 65 years, male gender, hypertension, cardiovascular disease, lung disease, diabetes mellitus, chronic renal insufficiency and end-stage renal disease, and activity of the AIIRD, but not its type, influenced the outcome of COVID-19. After adjusting for the comorbidities associated with severe COVID-19, the risk of admission to intensive care units and mortality among AIIRD patients were similar to the general population (2, 5–7, 9–11, 14).

Chronic corticosteroid therapy (equivalent to prednisone > 10 mg per day) was associated with severe COVID-19 and a higher risk of hospitalization (9). On the other hand, conventional synthetic (cs) and biological/targeted synthetic (b/ts) disease-modifying anti-rheumatic drugs (DMARDs), especially anti-cytokines, were not associated with severe COVID-19; some of the studies even suggested a protecting effect of DMARDs (8, 13). However, the influence of anti-CD20 B-cell depleting agents (e.g. rituximab) on the outcome of severe COVID-19 remained unclear. The potential role of csDMARDs and b/ts DMARDs in the management of severe COVID-19 was investigated and some of the trials even yielded positive results (15–18).

With the introduction of the severe acute respiratory syndrome coronavirus-2 (SARS-CoV-2) vaccinations, concerns were raised whether AIIRD patients can mount a protective immune response, and whether the vaccination may trigger a flare-up of the AIIRD. Studies showed that the vast majority of AIIRD patients developed a significant humoral response following the administration of the second dose of the mRNA vaccine against SARS-CoV-2 virus. Only minor adverse effects were reported and no apparent impact on AIIRD activity was noted (19, 20).

Nevertheless, many questions regarding COVID-19 in AIIRD patients remain unanswered, such as the pattern of spread as compared to the general population, the differential risk incurred by the rheumatic disease type, stage and duration, the use of various classes of DMARDs, the effect of COVID-19 on the AIIRD, and the long-term efficacy of the various vaccinations in preventing severe COVID-19.

Herein, we present data from the Israeli multi-center rheumatic COVID-19 registry, summarizing 21 months of the Israeli experience in AIIRD patients with SARS-CoV-2 infection. The aims of our study were to compare the pattern of COVID-19 spread in our cohort to the general Israeli population, to analyze the profile and outcomes of COVID-19 in AIIRD patients before and after the vaccination campaign (4 outbreaks), to define the factors associated with adverse outcomes (including hospitalization and death), to evaluate the impact of vaccinations on COVID-19 morbidity, and to investigate the influence of the viral disease on AIIRD activity 1-3 month after recovery.

## Methods

### Study design

At the beginning of the pandemic, we established a national registry of AIIRD patients diagnosed with COVID-19, based on voluntary reporting by the treating rheumatologists. All the members of the Israeli Association of Rheumatology were encouraged to participate and repeatedly reminded.

Thirteen referral rheumatologic centers from Northern and Central Israel (capturing 80% of the population) participated in this study. Data collection started on March 1^st^, 2020 and is continuously updated. For the current study, we analyzed the data collected from March 1^st^ 2020, to November 30^th^, 2021. The study was approved by the Ethics Committee of the participating institutions.

### Patients and data collection

The registry included adult patients with AIIRD diagnosed with COVID-19. Data was reported by the treating rheumatologists using an electronic case report form. The diagnosis of COVID-19 was made by a positive SARS-CoV-2 polymerase chain reaction (PCR) RNA test on a nasopharyngeal swab sample. The indications for SARS-CoV-2 PCR testing in Israel comprised clinical symptoms or exposure to a confirmed COVID-19 close contact. The AIIRD was diagnosed by the treating rheumatologist according to the updated guidelines. Patients without a validated diagnosis of COVID-19, or with incomplete data regarding the AIIRD and COVID-19 outcome, were excluded from the study. Duplicate records were identified by matching age, sex, AIIRD diagnosis, and date of COVID-19 diagnosis.

Case information included the following data:

1. Basic demographic data: age, sex, and ethnicity.

2. Clinical data regarding the rheumatic disease: AIIRD diagnosis and duration, systemic involvement (cardiac, respiratory, renal, or vascular including thromboembolic events and vasculitis), treatment (csDMARDs, b/ts DMARDs, corticosteroids use, dose and treatment duration- before COVID-19 diagnosis and during the acute illness), AIIRD activity in the last visit prior to COVID-19 diagnosis (as defined by physician global assessment (PhGA)).

3. Comorbidities known as risk factors for severe COVID-19: ischemic heart disease (IHD), congestive heart failure (CHF), cardiac arrhythmias requiring anticoagulant treatment, hypertension (HTN), lung disease, diabetes mellitus (DM), chronic renal failure (CRF), obesity, and smoking.

4. COVID-19: Diagnosis date (positive PCR test), illness severity (mild, moderate, and severe according to Israeli Ministry of Health (IMH) criteria), hospital admission, if required, and length of stay, treatment for the viral disease (including the need for supplemental oxygen or invasive ventilation), COVID-19-related complications, and COVID-19 outcomes (resolution of acute illness or death). Severe illness was defined by SpO2 <94% on room air, a respiratory rate of >30 breaths/min, PaO2/FiO2 <300 mm Hg, or lung infiltrates >50% on chest X-ray. For patients admitted to the hospital, laboratory results, including blood count, biochemical tests, and levels of C-reactive protein (CRP) were collected. The mRNA vaccine administration dates were recorded.

5. The status of the AIIRD after COVID-19 recovery: Outpatient follow-up was performed by the treating rheumatologists one to three months following COVID-19. AIIRD activity was determined based on PhGA and laboratory tests and was defined as yes (active) or no (non-active), by the treating rheumatologist. The epidemiologic data regarding the number of COVID-19 confirmed cases in Israel, the number of severe cases, and the rate of mortality among the general population per day and per week, were extracted from the data dashboard of the IMH.

### Study endpoints

The primary endpoints were hospitalization and death.

The secondary endpoints were:

1. “Negative hospitalization outcome”, which is a composite of prolonged hospitalization (length of hospitalization above median) and death.

2. AIIRD disease activity post-recovery of COVID-19.

### Statistical analysis

Categorical variables were summarized as frequency and percentage. Continuous variables were evaluated for normal distribution using histogram, Q-Q plot and Kolmogorov-Smirnov test. Normally distributed continuous variables were presented as mean and standard deviation (SD), while skewed variables were reported as median and interquartile range (IQR). Chi-squared test and Fisher’s exact test are used to study the associations between categorical variables and hospitalization, independent sample t test and Mann-Whitney test were applied to study the associations with continuous variables. Multiple variable logistic regression using backwards stepwise methods (Wald test) were used as criteria for removal and variables with p>0.1 were removed. The same methods were used to study the association between the patients’ characteristics and mortality. In order to identify subgroups of patients with increased risk for hospitalization, CHAID (chi-squared automatic interaction detection) algorithm was used. Length of hospitalization was categorized as median or below and above median. Negative hospitalization outcome was considered if the patient died during hospitalization or was hospitalized above median. The association between a negative hospitalization outcome and categorical variables was studied using chi-squared test and Fisher’s exact test. The association with continuous variables was studied with independent sample t test or Mann-Whitney test.

Factors that were found to be associated with negative outcomes were included in a multiple variable logistic regression. Odds ratio (OR) and a 95% confidence interval were reported. All statistical tests were 2-sided and p<0.05 was considered as statistically significant. SPSS and NCSS software were used for all statistical analysis (IBM SPSS statistical software, version 27, IBM corp., Armonk, NY, USA 2020 and NCSS statistical software, version 2021, NCSS, LLC, Kaysville, Utah, USA).

## Results

### COVID-19 pandemic in Israel

During the study period we experienced 4 outbreaks of COVID-19 pandemic. The Israeli population on 2020 counted around 9,217,000 persons. Up to April 30^th^, 2021, according to the data of the IMH, 838,481 Israelis had confirmed COVID-19; 17,403 patients had severe disease (2.08%) and 6363 died (0.75% case fatality rate; 36.6% of the patients with severe disease). From mid-March 2021, almost 2 months after the initiation of the vaccination campaign, a significant decline of newly confirmed COVID-19 cases was noticed, despite easing the restrictions and opening the schools and commerce.

The 4^th^ outbreak in Israel started by the end of June 2021, with the worldwide spread of the delta variant and 6 months after the start of the vaccination campaign. During the following 5 months, according to the data of the IMH, (until November 30^th^, 2021), 507,762 more cases of COVID-19 were diagnosed (a total of 1,346,243 cases since the beginning of the pandemic). Almost 1.1% (5569 patients) had severe COVID-19 and 1,763 more patients died (31.7% of the patients with severe COVID-19).

### COVID-19 in AIIRD patients in Israel before the vaccination campaign (the first 3 outbreaks)

During the period of March 1^st^ 2020 until April 30^th^, 2021, 298 AIIRD patients with COVID-19 were included in the registry (**Table 1**). Only 7.9% of the patients were diagnosed with COVID-19 during the 1^st^ outbreak (March-May 2020), 52.3% were diagnosed during the 2^nd^ outbreak (June-October 2020), and 39.7% during the 3^rd^ outbreak (November 2020 – April 2021). The demographic and clinical characteristics of the patients are detailed in **Table 1**. A significant proportion of patients (130, 43%) had systemic involvement secondary to the AIIRD. The lungs were the organ most frequently involved (39, 12.9%), followed by the kidneys (27, 8.9%) and the heart (23, 7.6%). Fifty-five patients (18.2%) had an evidence of AIIRD-related vasculopathy, such as a history of thromboembolic disease, mostly in patients with systemic lupus erythematosus (SLE), antiphospholipid syndrome, systemic sclerosis (SSc) and vasculitis (Behcet disease, Takayasu arteritis and Giant cell arteritis). AIIRD therapy included csDMARDs (176, 58.7%), b/tsDMARDs (136, 45.0%) and prednisone (120, 39.7%) (**Table 1**).

**Table 1:** Demographic and clinical characteristics of AIIRD patients in the Israeli Rheumatologic COVID-19 registry.

Variable*	1^st^-3^rd^ outbreaks n=302	4^th^ outbreak n=110	All n=412
Covid-19 outbreak			
First (March-May 2020)	24 (7.9)		
Second (June-October 2020)	158 (52.3)		
Third (November 2020-April 2021)	120 (39.7)		
Fourth (July-November 2021)		110	
**Age (years)** mean(SD)	53.7 (15.4)	50.1 (14.7)	52.8 (15.3)
**Female n (%)**	214 (70.8)	80 (72.7)	295 (71.6)
AIIRD diagnosis			
RA	100 (33.1)	29 (26.4)	129 (31.3)
SpA (including PsA and AS)	61 (20.2)	27 (24.5)	88 (21.4)
SLE	41 (13.6)	17 (15.5)	58 (14.1)
Other CTD (SSc, IIM, MCTD, etc.)	30 (9.9)	19 (17.3)	49 (11.9)
Vasculitis	32 (10.6)	10 (9.1)	42 (10.2)
FMF	19 (6.2)	2 (1.8)	21 (5.1)
Sarcoidosis	12 (4.0)	1 (0.9)	13 (3.2)
Other AIIRD	7 (2.3)	5 (4.5)	12 (2.9)
**Any systemic involvement**	130 (43.0)	42 (38.2)	172 (41.7)
Lung involvement	39 (12.9)	16 (14.5)	55 (13.3)
Cardiac involvement	23 (7.6)	7 (6.3)	30 (7.3)
Renal involvement	27 (8.9)	9 (8.2)	36 (8.7)
Vascular involvement	55 (18.2)	24 (21.8)	79 (19.2)
**AIIRD disease duration** (years) median (IQR) (N=295)	10 (5, 15)	7 (4,15)	10 (4,15)
Current immunomodulatory treatment			
Prednisone	120 (39.7)	33 (30)	153 (37.1)
csDMARDs	176 (58.3)	53 (48.2)	230 (55.8)
MTX	73 (24.2)	14 (12.7)	87 (21.1)
MMF/MPA	22 (7.3)	9 (0.82)	31 (7.5)
Biologic or targeted synthetic medication	136 (45.0)	62 (56.3)	198 (48.1)
Anti-cytokines*	32 (10.6)	35 (31.8)	67 (16.3)
Rituximab	24 (7.9)	16 (14.5)	40 (9.7)
Abatacept	5 (1.7)	4 (3.6)	9 (2.2)
JAK i	14 (4.6)	4 (3.6)	18 (4.4)
**Comorbidities**	194 (64.2)	71 (64.5)	265 (64.3)
More than 1 comorbidity	80 (26.5)	27 (24.5)	107 (26.0)
IHD	11 (3.6)	4 (2.7)	15 (3.6)
Hypertension	83 (27.5)	20 (18.2)	103 (25.0)
Diabetes Mellitus	56 (18.5)	14 (12.7)	70 (17.0)
Congestive heart failure	10 (3.3)	5 (4.5)	15 (3.6)
Chronic renal failure	19 (6.3)	5 (4.5)	24 (5.8)
Obesity	63 (20.9)	18 (16.4)	81 (20.0)
Chronic lung disease (COPD, asthma)	31 (10.3)	10 (9.1)	41 (10.0)
Atherosclerosis and AF	16 (5.3)	11 (10)	27 (6.6)
Smoking (ever)	13 (4.3)	8 (7.3)	21 (5.1)

*Categorical variables are presented as number (%). Normally distributed continuous variables are presented as mean ± SD and skewed continues variables are presented as median (IQR).

AIIRD, autoimmune inflammatory rheumatic disease; COVID-19, coronavirus disease 2019; n, number; SD, standard deviation; RA, rheumatoid arthritis; SpA, spondyloarthritis; PsA, psoriatic arthritis; AS, ankylosing spondylitis; SLE, systemic lupus erythematosus; CTD, connective tissue disease; SSc, systemic sclerosis; IIM, idiopathic inflammatory myopathy; MCTD, mixed connective tissue disease; FMF, familial Mediterranean fever; IQR, interquartile range; csDMARDs, conventional synthetic disease modifying anti-rheumatic drugs; MTX; methotrexate, MMF, mycophenolate mofetil; MPA, mycophenolic acid; JAKi, Janus-kinase inhibitors; IHD, ischemic heart disease; COPD, chronic obstructive pulmonary disease; AF, atrial fibrillation.

Almost two-thirds of the patients had at least one comorbidity (194, 64.2%) and 26.5% (80 patients) had 2 or more comorbidities. Active AIIRD was diagnosed in 44.7% of patients during the last clinic visit prior to COVID-19 diagnosis.

Most of the patients had a mild SARS-CoV-2 infection (196, 64.9%). 33 patients (10.9%) had a moderate COVID-19 course, and the remainder (73, 24.2%) had a severe disease.

A total of 161 patients (53.3%) were hospitalized (the demographic and clinical data are described in **Tables 2**–**4**). There were remarkable differences in the rate of hospitalization among COVID-19 AIIRD patients between the outbreaks: the highest rate was during the 1^st^ outbreak – 87.5%, compared to 53.2% during the 2^nd^ outbreak and 46.7% during the 3^rd^ outbreak.

**Table 2:** COVID-19 course, treatment and complications in Israeli AIIRD patients.

Variable *	1^st^-3^rd^ outbreaks n=302	4^th^ (Delta/vaccinated) outbreak n=110	All n=412
Severity n (%)			
Mild	196 (64.9)	79 (71.8)	274 (66.5)
Moderate	33 (10.9)	15 (13.6)	48 (11.7)
Severe	73 (24.2)	16 (14.5)	89 (21.6)
**Hospitalization**	161 (53.3)	29 (26.4)	190 (46.1)
**Intubation**	23 (7.6)	6 (5.5)	29 (7.0)
**Non-invasive oxygen supplementation**	53 (17.5)	16 (14.5)	69 (16.7)
**COVID-19 specific Tx**	109 (36.1)	34 (30.9)	143 (34.7)
Antiviral (remdesivir)	26 (8.6)	4 (3.6)	30 (7.3)
Convalescent plasma	12 (4.0)	2 (1.8)	14 (3.4)
Antibiotic tx.	34 (11.3)	11 (10.0)	45 (10.9)
HCQ	6 (2.0)	1 (0.9)	7 (1.7)
LMWH	71 (23.5)	20 (18.2)	91 (22.1)
Corticosteroids	74 (24.5)	22 (20.0)	96 (23.3)
Biologic medication (tocilizumab/baricitinib)	5 (1.7)	3 (2.7)	8 (1.9)
Casirivimab and imdevimab	5 (1.7)	14 (12.7)	19 (4.6)
Rheumatic Tx. discontinued	102 (33.8)	36 (32.7)	138 (33.5)
**COVID-19 complications**	52 (17.2)	13 (11.8)	65 (15.8)
Secondary bacterial infection	20 (6.6)	9 (8.2)	29 (7.0)
Myocarditis	6 (2.0)	3 (2.7)	9 (2.2)
Acute kidney injury	7 (2.3)	1 (0.9)	8 (1.9)
Thromboembolism	2 (0.7)	4 (3.6)	6 (1.5)
Pneumonia	67 (22.2)	6 (5.5)	73 (17.7)
Other complication	21 (7.0)	4 (3.6)	25 (6.1)
Days hospitalized median(IQR)	6 (2,14)	0 (0,3)	4 (0,14)
**Mortality**	27 (8.9)	7 (6.4)	34 (8.3)
**Disease activity before COVID-19 (N=400)**	134 out of 300 (44.7)	34 out of 100 (34)	168 (42.0)
**Disease activity after COVID-19** (N=344)	60 out of 265 (22.6)	25 out of 79 (31.6)	85 (24.7)

*Categorical variables are presented as number (%). COVID-19, coronavirus disease 2019; AIIRD, autoimmune inflammatory rheumatic disease; n, number; tx, treatment; HCQ, hydroxychloroquine; LMWH, low molecular weight heparin; IQR, interquartile range.

**Table 3:** Laboratory tests of AIIRD patients hospitalized for COVID-19.

Variable *	1^st^-3^rd^ outbreaks, n=138	4^th^ (Delta/vaccinated) outbreak
WBC 10e3/µL	6 (4.25-10.17)	4.8 (2.50-23.7)
Lymphocytes 10e3/µL n=136	0.8 (0.5-1.38)	0.9 (0.4-2.5)
Hb g/dL	12 (10.48-13.53)	13.1 (7.2-15.9)
Platelets 10e3/µL n=137	203 (151-284)	181 (34-249)
CRP mg/L n=134	11.75 (2.68-24)	13 (0.05-150)
ALT U/L n=122	28.5 (18.75-52.75)	29 (10-1036)
AST U/L n=113	32 (21-50)	44 (17-3175)
Ferritin ng/mL n=70	374 (132.25-991.25)	873 (124-4720)
D-dimer FEU n=89	0.93 (0.51-2.35)	2.2 (0.5-80)
Triglycerides mg/dL n=40	107.5 (44-179)	107.5 (82-286)
CPK U/L n=67	66 (37-138)	129 (15-9700)

*Variables are presented as median (IQR). AIIRD, autoimmune inflammatory rheumatic disease; COVID-19, coronavirus disease 2019; n, number; WBC, white blood cells; Hb, hemoglobin; CRP, C-reactive protein; ALT, alanine aminotransferase; AST, aspartate aminotransferase; CPK, creatine phosphokinase; IQR, interquartile range.

**Table 4:** Univariate analysis of demographic, clinical and COVID-19-related variables in regard to hospitalization.

	1^st^-3^rd^ outbreak			4^th^ outbreak		
Variable*	Hospitalized (n=161)	Not-hospitalized (n=141)	P-value	Hospitalized (n=29)	Not-hospitalized (n=81)	P-value
Covid-19 outbreak						
First (March-May 2020)	21 (87.5)	3 (12.5)	**0.001**	29 (26.4)	81 (73.6)	**<0.001**
Second (June-October 2020)	84 (53.2)	74 (46.8)				
Third (November 2020-April 2021)	56 (46.7)	64 (53.3)				
**Age (years)** mean(SD)	58.3 (14.3)	48.4(15)	**<0.0001**	**60.2 (13.3)**	**46.2 (13.3)**	**<0.001**
**Female (%)**	112 (69.6)	102 (72.3)	0.61	23 (79.3)	57 (70.4)	0.3
**AIIRD diagnosis**			**0.002**			**0.001**
RA	55 (34.2)	45 (31.9)		15 (51.7)	14 (17.3)	
SpA (including PsA and AS)	22 (13.7)	39 (27.7)		2 (6.9)	25 (30.9)	
SLE	17 (10.6)	24 (17.0)		4 (13.7)	13 (16.0)	
Other CTD (SSc, IIM, MCTD, etc.)	17 (10.6)	13 (9.2)		3 (10.3)	16 (19.7)	
Vasculitis	20 (12.4)	12 (8.5)		2 (6.9)	8 (9.9)	
FMF	15 (9.3)	4 (2.8)		2 (6.9)	0 (0)	
Sarcoidosis	11 (6.8)	1 (0.7)		0 (0)	1 (1.2)	
Other AIIRD	4 (2.5)	3 (2.1)		1 (3.4)	4 (4.9)	
**Any systemic involvement**	76 (47.2)	54 (38.3)	0.128	11 (37.9)	29 (35.8)	0.9
Lung involvement	32 (19.9)	7 (5.0)	**<0.0001**	6 (20.7)	10 (12.3)	0.3
Cardiac involvement	16 (9.9)	7 (5.0)	0.1	1 (3.4)	6 (7.4)	0.9
Renal involvement	17 (10.6)	10 (7.1)	0.322	3 (10.3)	6 (7.4)	0.7
Vascular involvement	30 (18.6)	25 (17.7)	0.8	5 (17.2)	19 (23.5)	0.5
**AIIRD disease duration** (years) median (IQR) (N=295)	10 (5,16)	8 (4,12)	0.075	10 (4,20)	6 (4,13)	0.2
Chronic rheumatic treatment						
Prednisone	78 (48.4)	42 (29.8)	**0.001**	13 (44.8)	20 (24.7)	**0.034**
Prednisone dose (mg) median (IQR)	5 (5,10)	5 (5,8.5)	0.449	5 (2,20)	5 (5,40)	0.17
csDMARDs	92 (57.1)	84 (59.6)	0.7	20 (69.0)	34 (42.0)	**0.041**
MTX	40 (24.8)	33 (23.4)	0.8	6 (20.7)	8 (9.9)	0.2
MMF/MPA	7 (4.3)	15 (10.6)	**0.036**	2 (6.9)	7 (8.6)	0.9
Biologic/targeted synthetic medication	61 (37.9)	75 (53.2)	**0.008**	17 (58.6)	44 (54.3)	0.8
Anti-cytokines	15 (9.3)	17 (12.1)	0.4	7 (24.1)	28 (34.6)	0.2
Rituximab	13 (8.1)	11 (7.8)	0.9	4 (13.8)	12 (14.8)	0.9
Abatacept	2 (1.2)	3 (2.1)	0.7	3 (10.3)	1 (1.2)	**0.02**
JAK i	12 (7.5)	2 (1.4)	**0.013**	3 (10.3)	1 (1.2)	**0.02**
**Comorbidities**	119 (73.9)	75 (53.2)	**<0.0001**	23 (79.3)	40 (49.4)	**0.011**
More than 1 comorbidity	59 (36.6)	21 (14.9)	**<0.0001**	12 (41.4)	15 (18.5)	**0.001**
IHD	9 (5.6)	2 (1.4)	0.054	2 (6.9)	2 (2.5)	0.2
Hypertension	57 (35.4)	26 (18.4)	**<0.001**	8 (27.6)	12 (14.8)	0.064
Diabetes Mellitus	44 (27.3)	12 (8.5)	**<0.001**	4 (13.7)	10 (12.3)	0.7
Congestive heart failure	9 (5.6)	1 (0.7)	**0.022**	3 (10.3)	2 (2.5)	0.064
Chronic renal failure	19 (11.8)	0 (0)	**<0.001**	4 (13.7)	1 (1.2)	**0.005**
Obesity	35 (21.7)	28 (19.9)	0.7	7 (24.1)	11 (13.6)	0.3
Chronic lung disease (COPD, asthma)	24 (14.9)	7 (5.0)	**0.005**	5 (17.2)	4 (4.9)	**0.006**
Atherosclerosis and AF	13 (8.1)	3 (2.1)	**0.021**	4 (13.7)	7 (8.6)	0.5
Smoking (ever)	6 (3.7)	7 (5.0)	0.6	3 (10.3)	5 (6.2)	0.13
Active disease before COVID-19	81 (50.3)	56 (39.7)	0.081	8 (27.6)	16 (19.7)	0.07

*Categorical variables are presented as number (%). Normally distributed continuous variables are presented as mean ± SD and skewed continues variables are presented as median (IQR).

COVID-19, coronavirus disease 2019; AIIRD, autoimmune inflammatory rheumatic disease; n, number; SD, standard deviation; RA, rheumatoid arthritis; SpA, spondyloarthritis; PsA, psoriatic arthritis; AS, ankylosing spondylitis; SLE, systemic lupus erythematosus; CTD, connective tissue disease; SSc, systemic sclerosis; IIM, idiopathic inflammatory myopathy; MCTD, mixed connective tissue disease; FMF, familial Mediterranean fever; IQR, interquartile range; csDMARDs, conventional synthetic disease modifying anti-rheumatic drugs; MTX; methotrexate, MMF, mycophenolate mofetil; MPA, mycophenolic acid; JAKi, Janus-kinase inhibitors; IHD, ischemic heart disease; COPD, chronic obstructive pulmonary disease; AF, atrial fibrillation.

COVID-19 treatment and complications are detailed in **Table 2**.

The mortality rate among the whole cohort in the first three outbreaks was 8.9%, and 38% among the patients with severe COVID-19 (**Table 2**).

In multivariate logistic regression analysis, only age, rheumatic disease-related pulmonary involvement, DM and treatment with prednisone, MMF, or JAKi were significantly associated with hospitalization (**Supplementary Table S1**).

In univariate analysis, older age, systemic involvement by the AIIRD, especially pulmonary, cardiac, renal and vascular, treatment with prednisone and/or b/ts DMARDs, comorbidities (ischemic heart disease [IHD], CHF, CRF, or lung disease) and coexistence of 2 or more comorbidities were associated with a higher mortality, similar to the risk factors for hospitalization (**Table 5**). In our cohort, sex, ethnicity, and type and duration of the AIIRD were not associated with a higher mortality.

**Table 5:** Univariate analysis of demographic, clinical and COVID-19-related variables in regard to mortality.

	1^st^-3^rd^ outbreaks			4^th^ outbreak		
Variable*	Dead N=27	Alive N=275	P-value	Dead N=7	Alive N=103	P value
Age (years) mean(SD)	63.3 (15.4)	52.7 (15.1)	**0.003**	68.1 (10.5)	48.8 (14.1)	**0.002**
Female N (%)	19 (70.4)	195 (70.9)	0.9	7 (100)	73 (70.9)	0.2
AIIRD diagnosis						
RA	9 (33.3)	91 (33.1)	0.103	5 (71.4)	24 (23.3)	0.078
SpA (including PsA and AS)	0 (0)	61 (22.2)		1 (14.3)	26 (25.2)	
SLE	5 (18.5)	36 (13.1)		0 (0)	17 (16.5)	
Other CTD (SSc, IIM, MCTD etc.)	6 (22.2)	24 (8.7)		0 (0)	19 (18.4)	
Vasculitis	4 (14.8)	28 (10.2)		0 (0)	10 (9.7)	
FMF	1 (3.7)	18 (6.5)		1 (14.3)	1 (1.0)	
Sarcoidosis	1 (3.7)	11 (4.0)		0 (0)	1 (1.0)	
Other AIIRD	1 (3.7)	6 (2.2)		0 (0)	5 (4.9)	
Active disease in last visit prior to COVID-19	16 (12)	117 (88)	0.154	4 (57.1)	20 (19)	**0.028**
AIIRD with any systemic involvement	17 (62.9)	130 (47.3)	**0.029**	4 (57.1)	38 (36.9)	0.2
Lung involvement	8 (29.6)	31 (11.3)	**0.013**	2 (28.6)	14 (13.6)	0.3
Cardiac involvement	7 (25.9)	16 (5.8)	**0.002**	1 (14.3)	6 (5.8)	0.3
Renal involvement	7 (25.9)	20 (7.3)	**0.005**	1 (14.3)	8 (7.7)	0.4
Vascular involvement	10 (37.0)	45 (16.4)	**0.016**	0 (0)	24 (23.3)	0.3
AIIRD disease duration (years) median (IQR)	10 (6,16)	10 (5,14)	0.3	15 (4,18)	7 (4,15)	0.4
Chronic rheumatic treatment						
Prednisone	19 (70.4)	101(36.7)	**0.001**	4 (57.1)	29 (28.2)	0.2
Prednisone dose (mg) median (IQR)	5 (5,15)	5 (5,10)	0.533	5 (0,10)	5 (0,40)	0.47
DMARD	15 (55.6)	161 (58.5)	0.8	4 (57.1)	50 (48.5)	0.9
MTX	6 (22.2)	67 (24.4)	0.8	2 (28.6)	12 (11.7)	0.2
MMF/MPA	3 (11.1)	19 (6.9)	0.4	1 (14.3)	8 (7.8)	0.5
Biologic/targeted synthetic medications	7 (25.9)	129 (46.9)	**0.037**	6 (85.7)	55 (53.4)	0.2
Anti-cytokines	6 (22.2)	26 (9.5)	0.054	3 (42.9)	32 (31.1)	0.7
Rituximab	4 (14.8)	20 (7.3)	0.3	0 (0)	16 (15.5)	0.7
Abatacept	0 (0)	5 (1.8)	1	1 (14.3)	3 (2.9)	0.2
JAKi	1 (3.7)	13 (4.7)	1	2 (28.6)	2 (1.9)	**0.012**
Comorbidities	20 (74.1)	171 (62.2)	0.299	5 (71.4)	58 (56.3)	0.7
More than 1 comorbidity	12 (44.4)	68 (24.7)	**0.027**	3 (42.8)	24 (23.3)	0.12
IHD	4 (14.8)	7 (2.5)	**0.011**	1 (14.3)	3 (2.9)	0.2
Hypertension	9 (33.3)	74 (26.9)	0.501	3 (42.8)	17 (16.5)	0.077
Diabetes Mellitus	6 (22.2)	50 (18.2)	0.604	2 (28.6)	12 (11.7)	0.2
Congestive heart failure	6 (22.2)	4 (1.5)	**<0.0001**	1 (14.3)	4 (3.9)	0.3
Chronic renal failure	5 (18.5)	14 (5.1)	**0.019**	2 (28.6)	3 (2.9)	**0.023**
Obesity	5 (18.5)	58 (21.1)	0.812	1 (14.3)	17 (16.5)	0.9
Chronic lung disease (COPD, asthma)	6 (22.2)	25 (9.1)	**0.044**	1 (14.3)	9 (8.7)	0.4
Atherosclerosis and AF	2 (7.4)	14 (5.1)	0.646	1 (14.3)	10 (9.7)	0.6
Smoking (ever)	2 (7.4)	11 (4.0)	0.3	0 (0)	8 (7.8)	0.9

*Categorical variables are presented as number (%). Normally distributed continuous variables are presented as mean ± SD and skewed continues variables are presented as median (IQR).

COVID-19, coronavirus disease 2019; AIIRD, autoimmune inflammatory rheumatic disease; n, number; SD, standard deviation; RA, rheumatoid arthritis; SpA, spondyloarthritis; PsA, psoriatic arthritis; AS, ankylosing spondylitis; SLE, systemic lupus erythematosus; CTD, connective tissue disease; SSc, systemic sclerosis; IIM, idiopathic inflammatory myopathy; MCTD, mixed connective tissue disease; FMF, familial Mediterranean fever; IQR, interquartile range; csDMARDs, conventional synthetic disease modifying anti-rheumatic drugs; MTX; methotrexate, MMF, mycophenolate mofetil; MPA, mycophenolic acid; JAKi, Janus-kinase inhibitors; IHD, ischemic heart disease; COPD, chronic obstructive pulmonary disease; AF, atrial fibrillation. Statistically significant P-values are bolded.

In multivariate logistic regression analysis, only older age, renal and vascular involvement by the AIIRD, CHF and treatment with prednisone or B-cell depleting therapy were associated with a higher mortality (**Supplementary Table S2**).

The results of white blood cell counts, hemoglobin, platelet counts and C-reactive protein (CRP) levels were reported in 138 out of the 160 hospitalized patients (**Table 3**). Higher white blood cell count, lower hemoglobin, and higher CRP levels at admission correlated with prolonged hospitalization (7 or more days) and increased mortality (univariate analysis). In logistic regression analysis, lower hemoglobin, higher white blood cell count, renal involvement by the AIIRD and CHF were also associated with prolonged hospitalization and increased mortality rate.

AIIRD activity was assessed at the first visit in the rheumatology clinic 1-3 months after recovery and reported for 265 patients. Prior to COVID-19, 44.7% of the patients had active AIIRD, while only 22.6% (60 out of 265 patients) displayed disease activity post-COVID-19; 42 of them (70%) also had active AIIRD pre-COVID-19. Most of them complained of diffuse musculoskeletal pain.

Notably, some of the patients, especially those with moderate to severe disease, discontinued the csDMARDs and/or the b/tsDMARDs during the viral illness, and many received short courses of corticosteroids for COVID-19.

### COVID-19 in AIIRD patients in Israel after the implementation of full-scale vaccination (the 4^th^ outbreak)

The 4^th^ outbreak started 6 months after the beginning of the vaccination campaign, with the spread of the delta variant. During the period of June 30^th^, 2021 till November 30^th^, 2021, 110 AIIRD patients with confirmed COVID-19 were included in the registry. The demographic data of the patients, the clinical characteristics of the AIIRDs, the immunomodulatory treatment and the comorbidities were similar to those in the previous outbreaks (**Table 1**). The majority of patients (76%) were vaccinated, however, more than 5 months elapsed from the second vaccine dose. Twenty two patients (20%) were diagnosed with COVID-19 within one week of the third (booster) dose.

Sixteen patients (14.5%) had severe COVID-19 (compared to 24.2% during the first 3 outbreaks). Twenty-nine patients (26.4%) were hospitalized (compared to 53.3% during the previous outbreaks). The OR for hospitalization in vaccinated patients compared to unvaccinated was 0.11 (0.01 – 0.63 95% CI, p=0.041) in those who received the vaccine within the prior 1-5 months, and 0.38 (0.21-0.67 95% CI, p=0.001) in those who received the vaccine more than 6 months before (**Tables 2**, **4**; **Supplementary Table S1**).

COVID-19 treatment and complications are detailed in **Table 2**.

Twenty-six patients (8.6%) were treated with remdesivir and 5 patients (1.7%) with monoclonal antibodies (casirivimab and imdevimab) during the first 3 outbreaks, compared to 4 (3.6%) and 14 (12.7%) patients respectively, during the delta outbreak. Due to the small number of patients who received these treatments, their real impact on COVID-19 morbidity or mortality was difficult to assess.

The mortality rate during the 4^th^ outbreak was significantly lower (6.4% vs 8.9%). Three out of the seven patients who died were more than 6 months after the 2nd mRNA vaccine; one patient received the booster at the same day of COVID-19 diagnosis, and two were unvaccinated. Only one patient was diagnosed with COVID-19 3 weeks after the booster dose and died. The latter patient suffered from familial Mediterranean fever, amyloidosis, and CRF (**Tables 5**; **Supplementary Table S2**).

AIIRD activity appeared comparable before and after contracting COVID-19 (**Table 2**). Most of the active patients complained of diffuse musculoskeletal pain.

**Figure 1:**
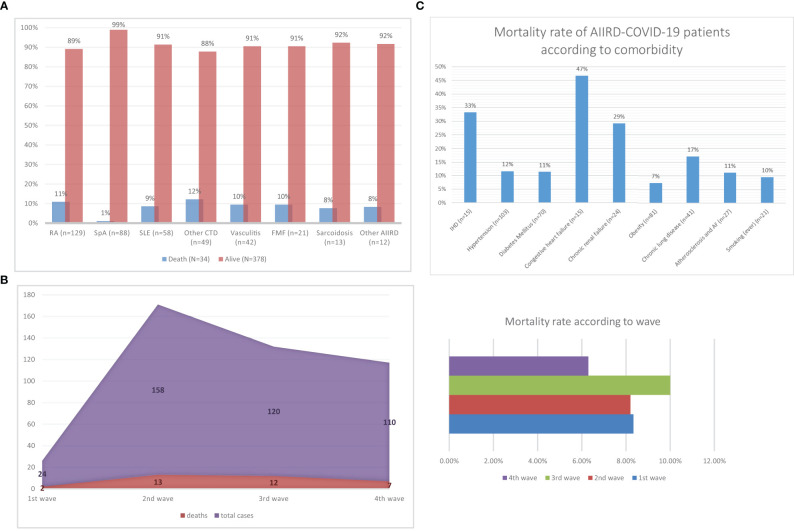
Mortality rate from COVID-19 according to AIIRD diagnosis, outbreak and comorbidities. 2 **(A)** Mortality rate according to AIIRD diagnosis. COVID-19, coronavirus disease 2019; AIIRD, autoimmune inflammatory rheumatic disease; n, number; RA, rheumatoid arthritis; SpA, spondyloarthritis; PsA, psoriatic arthritis; AS, ankylosing spondylitis; SLE, systemic lupus erythematosus; CTD, connective tissue disease; SSc, systemic sclerosis; IIM, idiopathic inflammatory myopathy; MCTD, mixed connective tissue disease; FMF, familial Mediterranean fever. 2 **(B)** Mortality (absolute number of cases) according to COVID-19 outbreaks. COVID-19, coronavirus disease 2019. 2 **(C)** Mortality rate of COVID-19 according to comorbidity. AIIRD, autoimmune inflammatory rheumatic disease; COVID-19, coronavirus disease 2019.

**Figures 1A–D** provide a detailed graphic presentation of the mortality rates of AIIRD COVID-19 patients during the different outbreaks.

**Supplementary Figures S1–3** detail the immunomodulatory drugs and the hospitalization rates of the patients.

### COVID-19 spread in AIIRD patients compared to the general population

**Figure 2:**
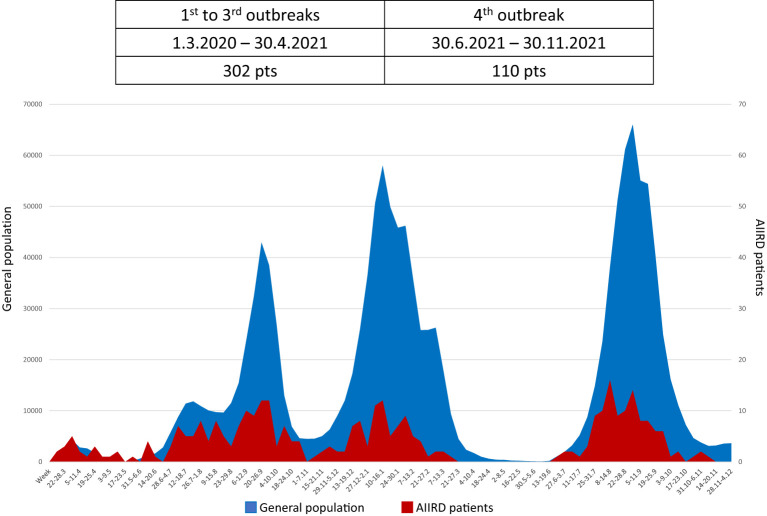
The pattern of spread of COVID 19 among AIIRD patients compared to the general population.

The weekly incidence curve of patients with AIIRD correlated with the curve of the general population (**Figure 2**). The pattern of spread of COVID-19 in AIIRD patients was similar to the general population.

## Discussion

During the study period, we experienced 4 outbreaks of COVID-19 pandemic. In December 2020, the IMH launched a national campaign to vaccinate the population with 2 doses of BNT162b2 (Pfizer-BioNTech) messenger RNA (mRNA) anti-SARS-CoV-2 vaccine, given 3 weeks apart. The first to be vaccinated were people older than 60 years of age, immunosuppressed patients, and medical personnel, and subsequently, the general population. This was followed by a gradual decrease in the age threshold to 12 years. By the end of April 2021, 55% of the population and almost 90% of people aged 50 years and more were fully vaccinated. By the end of July 2021, with the spread of the delta variant, the 2^nd^ vaccination campaign started with the administration of the 3^rd^ dose (booster vaccine), first to patients older than 60 years and the immunosuppressed patients, and then to the entire population older than 12 years of age.

In this study we report COVID-19 outcomes in AIIRD patients from 13 medical centers in Israel before and after the launching of the vaccination campaign.

Despite repeated mass media alerts for enhanced social distancing for the elderly and immunosuppressed, the pattern of COVID-19 spread in AIIRD patients was similar to the general population in Israel, throughout the entire period of time.

Although the demographic and clinical profiles of AIIRD patients who contracted COVID-19 were similar before and after the vaccination campaign, there were statistically significant differences in the hospitalization and mortality rates.

Prior to the vaccination era, the hospitalization rate was similar to the reported rate in the global rheumatology alliance (C19-GRA) physician registry and other registries (53.7% vs. 46% and 58% respectively) (9, 10, 14). The highest rate of hospitalization in our cohort was during the 1^st^ outbreak (87.5%), compared to 53.2% during the 2^nd^ outbreak and 46.7% during the 3^rd^. This might be explained by the experience gained in the management of COVID-19 patients throughout the pandemic, the changes in the criteria for hospitalization and ventilation support and the establishment of advanced home-care settings provided by the Israeli health maintenance organizations. The 8.9% mortality rate in our cohort in the first 3 outbreaks was similar to the rate reported by other registries (9, 10, 14). Severe COVID-19 led to a higher mortality rate among AIIRD patients as compared to the general population (38% vs 30%).

The risk factors for hospitalization and mortality among our cohort (prior to vaccinations) were comparable to those reported by other studies (5, 7, 9–12, 14) (older age, pulmonary involvement by the rheumatic disease, and DM for hospitalization; older age, renal and vascular involvement by the AIIRD, and CHF for mortality).

In our study, during the first 3 outbreaks, prior to the vaccination campaign, treatment with MMF or JAKi, but not anti-cytokines, was significantly associated with a higher risk of hospitalization, but not with mortality. Chronic prednisone treatment and B-cell depleting therapy were associated with a higher mortality. A similar association between JAKi treatment in rheumatoid arthritis patients and severe COVID-19 outcomes was reported by the GRA registry (13). These findings are somewhat intriguing, as one of the JAKi, baricitinib, was found to be beneficial in treating severe COVID-19. To our knowledge, most of the patients who contracted COVID-19 temporarily discontinued tsDMARDs. We may postulate that due to the relatively short half-life of JAKi, these agents could not provide a protective effect against the cytokine storm, unlike other bDMARDs, which have a longer half-life.

The “game changer” in the outcomes of COVID-19 among our patients was the vaccination campaign. The hospitalization and mortality rates were significantly lower during the 4^th^ outbreak, despite the fact that the delta variant, responsible for this outbreak, was considered to be more contagious and virulent. The main risk factor for hospitalization, besides older age, was the duration of time that elapsed after the second dose of COVID-19 mRNA vaccine: The OR for hospitalization of vaccinated patients compared to unvaccinated were 0.11(0.01 – 0.63 95% CI) in those who were 5 months and less after the 2^nd^ vaccine and 0.38 (0.21-0.67 95% CI), in those who received the vaccine more than 6 months before. Most of the cases of severe COVID-19 among our vaccinated patients occurred during the first 4-6 weeks of the 4^th^ outbreak, before the administration of the booster vaccine. Six out of the seven patients who died during this outbreak were more than 6 months after the 2^nd^ vaccine or were unvaccinated.

Despite the fact that MMF and rituximab were known to be associated with diminished humoral response to mRNA vaccines (19, 20), we did not find a correlation between treatment with MMF or rituximab (and JAKi) and increased rates of hospitalization or mortality in vaccinated patients.

Our study has some limitations. These include the observational and voluntary nature of the registry, that exposes it to reporting bias, potentialy increasing the estimation of adverse outcomes.

We do not have data regarding the impact of vaccination on the duration of COVID-19 symptoms or swab positivity.

Our study summarized data from the first 3 outbreaks and compared that to the 4th outbreak caused by the delta variant, which was considered more contagious and virulent. Patients with the omicron variant, which was associated with a milder clinical course, were not included in the time frame of our study.

The strenghts of our study include the large, diverse and representative, well-defined AIIRD patient population, the longitudinal data collection across four COVID-19 outbreaks from the start of the pandemic, the impact of the well-documented vaccinations and the follow up of patients by their treating rheumatologists post-COVID-19 recovery.

In conclusion, the epidemiology of COVID-19 in AIIRD patients is similar to the general population. The management of the patients, throughout the pandemic, was shifted from inhospital to home care, with the establishment of advanced home care settings provided by the Israeli health maintenance organizations. AIIRD characteristics, comorbidities, and drug treatment are identifiable risk factors for severe COVID-19. The vaccination against SARS-CoV-2 significantly reduced the rates of severe COVID-19, hospitalization and mortality. COVID-19 did not result in an increased proportion of AIIRD activity.

## Data availability statement

The raw data supporting the conclusions of this article will be made available by the authors, without undue reservation.

## Ethics statement

The studies involving human participants were reviewed and approved by the Ethics Committee of the participating institutions. Written informed consent for participation was not required for this study in accordance with the national legislation and the institutional requirements.

## Author contributions

FK and TE –collected clinical data, performed the data analysis and wrote the manuscript, MAB designed the database, performed the data analysis and contributed to writing, YZ contributed to the statistical analysis, MB, EP-S, KT, SO, ML, YT, MA-V, FS, GSB, AD, RB-G, DOM, ME, JF, OT-S, TG, TR, LR, SG, HP – diagnosed and treated the patients, collected clinical data and reviewed the manuscript. DP, DZ, ML, YM, OE, DM and AB-G treated the patients, designed the study and reviewed the manuscript. YBM – Initiated and designed the study, performed the data analysis, contributed to writing and edited the manuscript. All authors contributed to the article and approved the submitted version.
