# Supplementary material for: A deep look into the storm: Israeli multi-center experience of coronavirus disease 2019 (COVID-19) in patients with autoimmune inflammatory rheumatic diseases before and after vaccinations
Source: Front Immunol. 2023 Mar 13;14:1064839. doi: 10.3389/fimmu.2023.1064839 (PMC10040776; doi:10.3389/fimmu.2023.1064839)
Supplement: Supplementary file 1 [file DataSheet_1.docx]

**A deep look into the storm: Israeli multi-center experience of coronavirus disease 2019 (COVID-19) in patients with autoimmune inflammatory rheumatic diseases before and after vaccinations**

Fadi Kharouf¹^,^³*, Tali Eviatar²^,^⁴*, Maya Braun³, Elisheva Pokroy-Shapira⁴^,^⁵, Michal Brodavka^4,6^, Yair Zloof^3^, Nancy Agmon-Levine⁴^,7^, Kochava Toledano^8,9^ , Shirly Oren⁴^,^⁵, Merav Lidar⁴^,^⁶, Devy Zisman^9,10^ , Yonit Tavor^8,9^, Mirit Amit-Vazina⁴^,11^, Firas Sabbah^12^, Gabriel S. Breuer^3,13^ , Amir Dagan^4,14^, Rima Beshara-Garzuzi^9,10^ , Doron Markovits^8,9^, Muna Elias^9,10^, Joy Feld^9,10^, Oshrat Tayer-Shifman^4,15^ , Tal Gazitt^9,10^_,_ Tatiana Reitblatt^16^, Limor Rubin^3,17^, Amir Haddad^9,10^, Sami Giryes^8,9^, Daphna Paran²^,^⁴, Hagit Peleg ¹^,^³, Yair Molad ⁴^,^⁵, Ori Elkayam²^,^⁴, Dror Mevorach¹^,^³ Alexandra Balbir-Gurman^8,9^ and Yolanda Braun-Moscovici^8,9^

¹The Department of Medicine, Rheumatology Unit and Rare Disease Research Center, Hadassah Medical Center, Jerusalem, ² Rheumatology Department, Sourasky Medical Center, Tel Aviv, ³ The Faculty of Medicine, Hebrew University of Jerusalem, ⁴ Sackler Faculty of Medicine, Tel Aviv University, ⁵Institute of Rheumatology, Rabin Medical Center, Beilinson Hospital, Petach Tikva, ⁶Rheumatology Unit, Sheba Medical Center, Tel HaShomer, ⁷Clinical Immunology, Angioedema and Allergy Unit, Sheba Medical Center, Tel HaShomer, ^8^Rheumatology Institute, Rambam Health Care Campus, Haifa, ^9^Rappaport Faculty of Medicine, Israeli Institute of Technology-Technion, Haifa, ^10^ Rheumatology Unit, Carmel Medical Center, Haifa_,_ ^11^Rheumatology Service, Shamir Medical Center, Be'er Ya'akov, ^12^Rheumatology Service, Baruch Padeh Medical Center, Poriya, ^13^Rheumatology Unit, Shaare Tzedek Medical Center, Jerusalem, ^14^ Assuta Ashdod Hospital, Ashdod, ^15^Rheumatology Unit, Meir Medical Center, Kfar Saba, ^16^Barzilai Medical Center, Ashkelon, Ben Gurion University ^17^Allergy and Clinical Immunology Unit, Department of medicine, Hadassah Medical Center, Jerusalem.

**Supplementary File**

**Supplementary Figure S1**. Immunomodulatory medications used by AIIRD COVID-19 patients.


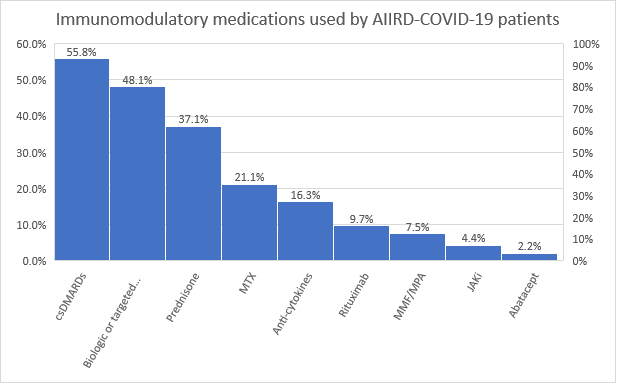


AIIRD, autoimmune inflammatory rheumatic disease; COVID-19, coronavirus disease 2019; csDMARDs, conventional synthetic disease modifying anti-rheumatic drugs; MTX; methotrexate, MMF, mycophenolate mofetil; MPA, mycophenolic acid; JAKi, Janus-kinase inhibitors

**Supplementary Figure S2**. Hospitalization and ambulatory rate of AIIRD COVID-19 patients according to COVID-19 outbreak.

P=0.001

COVID-19, coronavirus disease 2019; AIIRD, autoimmune inflammatory rheumatic disease; n, number.

**Supplementary Figure S3**. Hospitalization and ambulatory rate of AIIRD COVID-19 patients according to AIIRD diagnosis.

AIIRD, autoimmune inflammatory rheumatic disease; COVID-19, coronavirus disease 2019; n, number; RA, rheumatoid arthritis; SpA, spondyloarthritis; PsA, psoriatic arthritis; AS, ankylosing spondylitis; SLE, systemic lupus erythematosus; CTD, connective tissue disease; SSc, systemic sclerosis; IIM, idiopathic inflammatory myopathy; MCTD, mixed connective tissue disease; FMF, familial Mediterranean fever.

**Supplementary Table S1**. Backward stepwise logistic regression final model for prediction of hospitalization of AIIRD patients with COVID-19*.

***1A -* 1^st^-3^rd^ outbreaks**

| Variable | OR | 95% CI | p-value |
| --- | --- | --- | --- |
| Age, for each year | 1.04 | 1.02-1.06 | **<0.001** |
| AIIRD Lung involvement | 6.37 | 2.48-19.5 | **<0.001** |
| Prednisone tx | 1.95 | 1.12-3.43 | **0.018** |
| MMF/MPA | 0.27 | 0.08-0.81 | **0.026** |
| JAKi | 3.66 | 0.90-24.7 | 0.11 |
| Diabetes Mellitus | 2.64 | 1.28-5.73 | **0.011** |

* Variables entered on step 1 included district, age, AIIRD lung involvement, AIIRD cardiac involvement, Prednisone treatment, MMF/MPA treatments, biologic medication treatment, JAKi treatment, AIIRD disease duration, IHD, hypertension, diabetes mellitus, congestive heart failure, chronic lung disease (COPD, asthma), atherosclerosis and AF.

AIIRD, autoimmune inflammatory rheumatic disease; COVID-19, coronavirus disease 2019; OR, odds ratio; CI, confidence interval; tx, treatment; MMF, mycophenolate mofetil; MPA, mycophenolic acid; JAKi, Janus-kinase inhibitors; IHD, ischemic heart disease; COPD, chronic obstructive pulmonary disease; AF, atrial fibrillation.

**1B – 4^th^ outbreak**

| Variable | OR | 95% CI | P-value |
| --- | --- | --- | --- |
| Age | 1.09 | 1.04-1.14 | **<0.001** |
| AIIRD lung involvement | 1.22 | 0.26-5.23 | 0.8 |
| Prednisone tx | 2.63 | 0.76-9.67 | 0.13 |
| Chronic renal failure | 16.8 | 1.79-156.91 | **0.013** |
| csDMARDs | 3.49 | 1.12-12.4 | **0.038** |
| Time after 2^nd^ vaccine <6 months | 0.11 | 0.01-0.63 | **0.041** |
| Time after 2^nd^ vaccine ≥6 months | 0.38 | 0.21-0.67 | **0.001** |

* variables entered on step 1 included district, age, AIIRD lung involvement, AIIRD cardiac involvement, Prednisone treatment, MMF/MPA treatments, biologic medication treatment, JAKi treatment, AIIRD disease duration, IHD, hypertension, diabetes mellitus, congestive heart failure, chronic lung disease (COPD, asthma), atherosclerosis and AF.

AIIRD, autoimmune inflammatory rheumatic disease; COVID-19, coronavirus disease 2019; OR, odds ratio; CI, confidence interval; tx, treatment; csDMARDs, conventional synthetic disease modifying anti-rheumatic drugs; MMF, mycophenolate mofetil; MPA, mycophenolic acid; JAKi, Janus-kinase inhibitors; IHD, ischemic heart disease; COPD, chronic obstructive pulmonary disease; AF, atrial fibrillation.

**Supplementary Table S2**. Backward stepwise logistic regression final model for prediction of mortality of AIIRD patients with COVID-19*.

**2A – 1^st^-3^rd^ outbreaks**

| Variable | OR | 95% CI | p-value |
| --- | --- | --- | --- |
| Age, for each year | 1.062 | 1.021-1.096 | **0.001** |
| AIIRD renal involvement | 6.015 | 1.764-20.518 | **0.004** |
| AIIRD vascular involvement | 3.249 | 1.149-9.187 | **0.026** |
| Prednisone tx | 3.019 | 1.088-8.383 | **0.034** |
| B cell targeted therapy | 3.736 | 1.081-12.911 | **0.037** |
| Congestive heart failure | 12.025 | 2.324-62.225 | **0.003** |

* Variables entered on step 1 included district, age, AIIRD systemic involvement, AIIRD lung involvement, AIIRD cardiac involvement, AIIRD kidney involvement, AIIRD vascular involvement, Prednisone treatment, biologic/targeted synthetic medications, B cell targeted therapy, ischemic heart disease, congestive heart failure, chronic kidney disease, chronic lung disease (COPD, asthma), atherosclerosis and AF.

AIIRD, autoimmune inflammatory rheumatic disease; COVID-19, coronavirus disease-19; OR, odds ratio; CI, confidence interval; tx, treatment; MMF, mycophenolate mofetil; MPA, mycophenolic acid; JAKi, Janus-kinase inhibitors; IHD, ischemic heart disease; COPD, chronic obstructive pulmonary disease; AF, atrial fibrillation.

**2B – 4^th^ outbreak**

| Variable | OR | 95% CI | p-value |
| --- | --- | --- | --- |
| Age | 1.11 | 1.04-1.21 | **0.007** |
| Chronic renal failure | 14 | 1.13-208 | **0.037** |

* Variables entered on step 1 included district, age, AIIRD systemic involvement, AIIRD lung involvement, AIIRD cardiac involvement, AIIRD kidney involvement, AIIRD vascular involvement, Prednisone treatment, biologic/targeted synthetic medications, B-cell targeted therapy, ischemic heart disease, congestive heart failure, chronic kidney disease, chronic lung disease (COPD, asthma), atherosclerosis and AF.

AIIRD, autoimmune inflammatory rheumatic disease; COVID-19, coronavirus disease 2019; OR, odds ratio; CI, confidence interval; tx, treatment; csDMARDs, conventional synthetic disease modifying anti-rheumatic drugs; MMF, mycophenolate mofetil; MPA, mycophenolic acid; JAKi, Janus-kinase inhibitors; COPD, chronic obstructive pulmonary disease; AF, atrial fibrillation.
